# A Telehealth Intervention Using Nintendo Wii Fit Balance Boards and iPads to Improve Walking in Older Adults With Lower Limb Amputation (Wii.n.Walk): Study Protocol for a Randomized Controlled Trial

**DOI:** 10.2196/resprot.4031

**Published:** 2014-12-22

**Authors:** Bita Imam, William C Miller, Heather C Finlayson, Janice J Eng, Michael WC Payne, Tal Jarus, Charles H Goldsmith, Ian M Mitchell

**Affiliations:** ^1^Graduate Program in Rehabilitation SciencesUniversity of British ColumbiaVancouver, BCCanada; ^2^Department of Occupational Science and Occupational TherapyUniversity of British ColumbiaVancouver, BCCanada; ^3^Division of Physical Medicine and RehabilitationUniversity of British ColumbiaVancouver, BCCanada; ^4^Department of Physical TherapyUniversity of British ColumbiaVancouver, BCCanada; ^5^Department of Physical Medicine and RehabilitationWestern UniversityLondon, ONCanada; ^6^Faculty of Health SciencesSimon Fraser UniversityBurnaby, BCCanada; ^7^Department of Computer ScienceUniversity of British ColumbiaVancouver, BCCanada

**Keywords:** amputation, adult, aged, randomized controlled trial, telemedicine, walking

## Abstract

**Background:**

The number of older adults living with lower limb amputation (LLA) who require rehabilitation for improving their walking capacity and mobility is growing. Existing rehabilitation practices frequently fail to meet this demand. Nintendo Wii Fit may be a valuable tool to enable rehabilitation interventions. Based on pilot studies, we have developed “Wii.n.Walk”, an in-home telehealth Wii Fit intervention targeted to improve walking capacity in older adults with LLA.

**Objective:**

The objective of this study is to determine whether the Wii.n.Walk intervention enhances walking capacity compared to an attention control group.

**Methods:**

This project is a multi-site (Vancouver BC, London ON), parallel, evaluator-blind randomized controlled trial. Participants include community-dwelling older adults over the age of 50 years with unilateral transtibial or transfemoral amputation. Participants will be stratified by site and block randomized in triplets to either the Wii.n.Walk intervention or an attention control group employing the Wii Big Brain cognitive software. This trial will include both supervised and unsupervised phases. During the supervised phase, both groups will receive 40-minute sessions of supervised group training three times per week for a duration of 4 weeks. Participants will complete the first week of the intervention in groups of three at their local rehabilitation center with a trainer. The remaining 3 weeks will take place at participants’ homes using remote supervision by the trainer using Apple iPad technology. At the end of 4 weeks, the supervised period will end and the unsupervised period will begin. Participants will retain the Wii console and be encouraged to continue using the program for an additional 4 weeks’ duration. The primary outcome measure will be the “Two-Minute Walk Test” to measure walking capacity. Outcome measures will be evaluated for all participants at baseline, after the end of both the supervised and unsupervised phases, and after 1-year follow up.

**Results:**

Study staff have been hired and trained at both sites and recruitment is currently underway. No participants have been enrolled yet.

**Conclusions:**

Wii.n.Walk is a promising in-home telehealth intervention that may have useful applications for older adults with LLA who are discharged from rehabilitation or live in remote areas having limited or no access to existing rehabilitation programs.

**Trial Registration:**

Clinicaltrial.gov NCT01942798; http://clinicaltrials.gov/ct2/show/NCT01942798 (Archived by WebCite at http://www.webcitation.org/6V0w8baKP).

##  Introduction

In 2003, it was estimated that more than 2 million individuals were living with lower limb amputation (LLA) in North America with an annual incidence of 150,000 [1]. Over half of LLAs are transtibial (TT) and transfemoral (TF) amputations [2]. In Western countries, the incidence of LLA increases sharply after the age of 50 years as a result of secondary complications associated with illnesses such as diabetes and vascular disease [2].

Recovery following LLA is notably slow. A lengthy recovery process is especially common among older adults who often have multiple co-morbidities including peripheral vascular disease, peripheral neuropathy, hypertension, heart disease [3,4], and cognitive impairment [5]. LLA, compounded with these co-morbidities, influences walking and places these individuals at a high risk of falling and sustaining injury after a fall [6]. In fact, 52% of community-dwelling individuals with LLA report falling each year [7]. Similarly, 49% have a fear of falling and 65% report low balance confidence [7]. The consequences associated with these numbers may contribute to deterioration in balance [8], endurance, strength, and coordination [9] in older adults, and ultimately a decline in walking capacity. Walking capacity is a strong determinant of health-related quality of life (HRQOL) in individuals with LLA [10-13] as well as the best predictor of prosthetic walking in individuals with LLA [14]. The ability to walk longer distances allows the individual to move around his or her environment independently, which in turn impacts one’s choice of activities and participation [15].

Following an LLA, individuals need to participate in prosthetic rehabilitation. Rehabilitation includes procurement of a prosthetic limb and ambulation training. The costs associated with post-amputation care and prosthetic rehabilitation are considerable. Post-LLA, projected lifetime health care costs total approximately US $509,000 [16]. Due to escalating costs, existing rehabilitation programs are experiencing difficulty providing sufficient levels of prosthetic therapy [1]. Furthermore, a trend toward outpatient rehabilitation, community inaccessibility, and transportation barriers imposes challenges for clients, particularly those in rural/remote areas, to attend face-to-face clinic appointments [17]. Therefore, accessible and innovative approaches are needed to improve outcomes for individuals with LLA and overcome these barriers to participation.

In-home telehealth is an innovative and emerging approach to provide rehabilitation through technologies and telecommunication [17-20]. Home treatments create accessible rehabilitation programs, promote continuity of care after discharge, and offset the time and expense of travel for clients to in-hospital rehabilitation programs [21,22]. Access to in-home rehabilitation is particularly important for those with limited access to facilities and transportation [23].

Nintendo Wii Fit is a commercial gaming technology that shows promise as an in-home rehabilitation tool. The benefits of using Wii Fit technology as a rehabilitation tool are demonstrated by the growing knowledge base on the use of gaming technology in older adult rehabilitation. In a study of older adults that used Wii Fit during in-patient rehabilitation, more than 80% expressed their desire to continue using Wii Fit at home [24]. Preliminary evidence suggests Wii Fit training is a feasible and safe method leading to improvements in balance [25,26], walking [25-27], and balance confidence in older adults [27]. Studies have reported improvements in walking and balance confidence in individuals with multiple sclerosis [28] and improved balance and decreased risk of falls in individuals with mild Alzheimer’s [29]. Pilot testing has shown improvements in balance, balance confidence, and gait variables [25] in two older adults with LLA, which is consistent with findings from our own pilot work [30].

In a Single Subject Research Design (SSRD) study of six individuals with LLA, the feasibility of a Wii Fit oriented intervention consisting of structured daily training varying from 2 to 6 weeks was assessed. Results indicated a statistical improvement in walking capacity in five participants who had 3 or more weeks of intervention [30]. The aim of the present study is to extend findings on this topic and conduct a randomized controlled trial (RCT) to assess our in-home telehealth Wii Fit intervention protocol we call “Wii.n.Walk” *(*Clinicaltrial.gov NCT01942798).

The primary clinical hypothesis is that participants in the Wii.n.Walk intervention group will experience an improvement in walking capacity compared to the control group. The secondary clinical hypothesis is that participants in the Wii.n.Walk intervention group will experience an improvement in lower limb functioning (balance, gait speed, and strength), dynamic balance, physical activity, and balance confidence. The tertiary clinical hypothesis suggests that the Wii.n.Walk group will experience an improvement in life space mobility, prosthetic use, HRQOL, and will have a lower incidence of falls. The adherence hypothesis is that the Wii.n.Walk group will have ≥80% adherence.

## Methods

### Trial Design

A parallel, evaluator-blind RCT conducted at two sites (Vancouver, British Columbia and London, Ontario) will be used. To minimize participants’ bias associated with knowing which intervention is of interest to researchers, we will attempt to mask participants to the true study objectives (NCT01942798). This will be achieved thorough stating that “evidence suggests that having good cognition improves physical outcomes and we are trying to determine whether cognitive or activity training is better” both in the consent form and when addressing subjects’ comments/questions.

**Figure 1:**
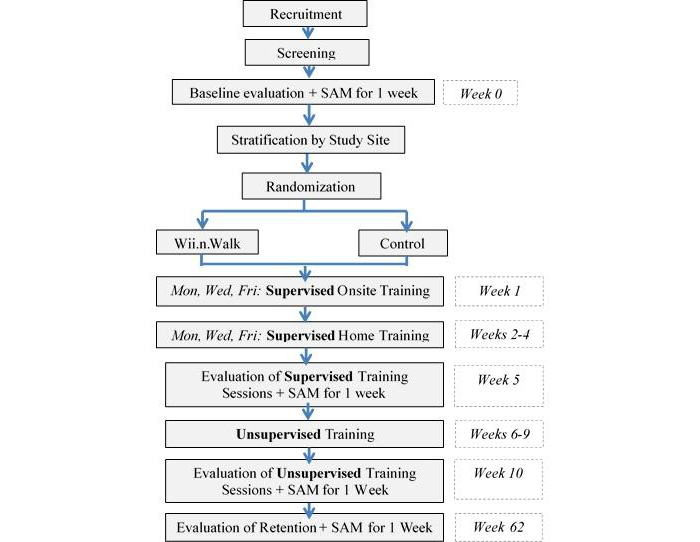
Study Flowchart.

Participants will be stratified by site and block randomized to the Wii.n.Walk intervention or control group in triplets using a 1:1 allocation ratio. To ensure balance between groups and masking of group assignment, a central computerized randomization process will be designed by the research team statistician, with undisclosed variable block sizes. Randomization will occur after the participant is screened and enrolled (Figure 1). The site coordinators will contact the statistician via telephone or email and obtain group assignment. The participant’s contact information will be forwarded to the appropriate group trainer to arrange for an initial training session.

This trial includes a supervised and an unsupervised phase for both the Wii.n.Walk intervention and control group. Once enrollment has been completed, participants will be evaluated on a number of clinical measures at baseline, after the supervised phase is completed, after the unsupervised phase is completed, and at a 1-year follow-up time point.

### Participants

A total of 72 community-dwelling prosthetic ambulators in London and Vancouver will be recruited through clinicians and prosthetists. A letter of information will be distributed to all individuals in the amputee program databases who meet the study inclusion criteria. Participants need to be ≥50 years of age, have a unilateral TT or TF amputation, use their prosthesis for at least 2 hours per day for the past 6 months to minimize the influence of residual limb/prosthetic fit problems, be cognitively able to engage in the program (receive a score on the Modified Mini-Mental Status Exam score of >23) [31], and have a television that will enable connection to the Nintendo hardware.

Individuals will be excluded if they cannot communicate in English, cannot provide informed consent, have medical conditions (eg, congestive heart failure) that limit exercise participation as determined using the American College of Sports Medicine exercise guidelines for older adults [32], have prosthetic fit issues (eg, pain and discomfort) as indicated by scores <6 on the Prosthetic Socket Fit Comfort Scale [33], or are currently participating in another supervised exercise or training program (eg, balance training).

Participants between 50-69 years old will be medically screened by the site coordinators using the Physical Activity Readiness Questionnaire (PAR-Q) [34]. Participants who are 70 or more years old or who answer “yes” to any of the PAR-Q questions will be medically screened by a physician using the Physical Activity Readiness Medical Examination (PARmed-X) to obtain clearance for physical activity participation [35].

### Wii.n.Walk Intervention

Participants in the experimental group will receive the Wii.n.Walk intervention. The Wii.n.Walk intervention was developed by core members of the research team and refined based on observations and the feedback received from the participants in the pilot studies [30]. Modifications for trainer instructions were made to Wii Fit postures and activities to prevent incorrect postures/techniques and to promote function and safety. Preliminary work also informed the dosage/frequency and duration of the intervention.

Social Cognitive Theory (SCT) [36] is the theoretical foundation for the Wii.n.Walk intervention. This theory was developed to enhance all four sources of self-efficacy: performance mastery, vicarious learning, verbal persuasion, and reinterpretation of physiological responses. Performance mastery, or learning to perform a specific skill, is the most robust source of self-efficacy. Successful performance of the Wii.n.Walk activities may provide a sense of accomplishment and thereby improve self-efficacy. Vicarious learning, or learning by watching others successfully accomplish activities, provides the observer a sense that they, too, have the ability to accomplish the task. This will be established by performing the Wii.n.Walk activities in groups initially and having participants watch the other group members perform the activities. Verbal persuasion will arise from credible feedback, guiding the learner through the task, and motivating his or her best effort. The trainer will provide this feedback when appropriate—at least once each session for each group member. The Nintendo device also automatically provides auditory and visual feedback based on the participant’s performance. Finally, participants will be taught to reinterpret physiological responses (eg, stress and anxiety) that may be associated with challenging Wii.n.Walk activities. According to a systematic review, physical activity programs that incorporate SCT are more effective in enhancing adherence [37]. More specifically, social support, peer modeling, and group training have been identified as important factors for increasing adherence in older adults [38].

The Wii.n.Walk protocol consists of Wii Fit activities. Participants stand on the Wii Fit balance board and play the games through weight shifting or by using the Wii remote control. The intervention protocol includes selected activities and exercises including yoga (static and dynamic single and double leg poses), balance tasks (lateral, posterior, and anterior weight shifting exercises), strength training (dynamic single and double leg exercises), and aerobics (running on the spot and step class).

Participants will complete two training phases: a 4-week supervised phase (3 times/week, 40 minutes training /session) followed by a 4-week unsupervised phase. Based on our pilot studies, completion of 40-minute Wii Fit exercises in each session takes approximately 60-90 minutes for the participants, including the instructions given by the Nintendo software as well as the rests that may be needed between the exercises. Participants will meet in groups of three at their local rehabilitation center with an experienced trainer during the first week to learn the program. They will then complete the final 3 weeks of the supervised program at home while being remotely supervised by the trainer and remotely interacting with the other two group participants. During the supervised phase, the trainer will provide individualized intervention and advance the training as the participant improves. At the end of the 4-week supervised phase, participants will retain the Wii units and be encouraged to use the program on their own for an additional 4 weeks (unsupervised phase).

The supervised phase purposely begins with in-person visits to introduce participants to the program, initiate group dynamics (eg, peer support), and create familiarity with the activities in a monitored safe environment. Graduating to home sessions overcomes barriers, cost, and inconveniences associated with travelling to a rehabilitation clinic and is intended to reinforce continued participation [22]. Supervised phase home sessions will be monitored by a trainer remotely using iPads with Wi-Fi plus cellular (Apple Inc, Cupertino, California, USA), preloaded with the VidyoMobile videoconferencing application (Vidyo Inc, Hackensack, New Jersey, USA). VidyoMobile enables the participant to meet at home with the trainer and the other two participants in the group. For better sound quality, participants will be asked to wear wireless headphones (Kinivo, Bellevue, Washington USA) with noise cancellation features. The iPad interface has been simplified as much as possible. Only the VidyoMobile app is available to the participants (access to all other apps is disabled through the iPad’s parental control feature), and they can connect to the trainer by entering only their names and a simple PIN code. iPads will be securely mounted on a sturdy tablet tripod and will be placed a few meters away from the participant’s TV and behind the participant, so that the trainer can see both the participant’s screen as well as his or her posture. The ideal location will be established by the trainer during the home setup of the Wii.n.Walk equipment. At the beginning of each session, the trainer will hold a brief discussion session with all participants, through the videoconferencing software, to review the plan for the session and address any questions. Once the session begins, the trainer is able to watch and supervise all three participants from his or her desktop/laptop at the clinic. The participant’s iPad can display the trainer and the other two participants. The trainer can remotely deactivate each iPad’s camera to reduce distractions while exercising or to reduce video streaming costs. The trainer will activate the iPad’s camera on at least two occasions during each session to enable opportunities for vicarious learning and participant-to-participant verbal persuasion. As an example, the trainer will ask two participants to watch the third perform an exercise. Verbal persuasion will be provided by the trainer to the participant being watched.

The exercises/games and their difficulty levels will be chosen by the trainer. The three participants in the same group will perform similar sets of exercises/games; however, the difficulty level of the exercises/games will vary depending on each participant’s abilities. By default, the more challenging levels of the games are initially locked and can only be unlocked if the participant successfully completes easier, prerequisite levels. In addition, progression to more difficult and longer activities is guided by instructions in the Wii.n.Walk manual. The manual also provides modified activity positions such as adding unilateral or bilateral external hand support if required by the participant. Modifications can be made if the participant has difficulty or is unable to do the activity. As an example, activities may be modified for an individual with a TF amputation if the prosthesis is not structurally capable of assuming the exercise position (eg, some of the exercises require stance phase prosthetic knee flexion). Common postural mistakes are included in the manual to guide the trainer in correcting positioning.

At the end of the supervised phase, the iPads, stands, and headphones will be collected by the trainer, while participants will retain the Wii console and balance board for the unsupervised phase. Participants will be encouraged to use the Wii.n.Walk program as much as they like during the unsupervised phase by continuing to do the same exercises/games they did during the supervised phase and progressing to the challenging levels if they unlocked those levels*.* To avoid confounding information, other people living with the participant will be asked not to use it. The trainer will telephone the participant once a week to monitor for safety (eg, falls) and equipment function.

For both supervised and unsupervised phases, depending on the level of ability and potential for safety issues, participants will be asked to have two high-back chairs placed on either side of them to minimize the risk of falling while using the Wii.n.Walk program. For participants who require additional assistance, if available, a family member, friend, or caregiver will be encouraged to be present during the training sessions. As the participant’s abilities improve, they will progress from the use of such assistance.

### Control Intervention

The control group will follow the same protocol but will be trained to use the Wii Big Brain Academy Degree program (Nintendo, Kyoto, Japan). Big Brain is a low-cost, commercially available software consisting of video games to improve cognitive function. Participants will use the Wii remote to participate in the games by pointing and clicking to select answers in response to on-screen questions. Big Brain games require participants to identify, memorize, analyze, compute, and visualize. The games have easy, medium, and difficult levels. Participants initially start with easy games and progress to more challenging levels based on their performance. The trainer will design the supervised sessions, provide instruction/feedback, and facilitate group discussions. Results from the feasibility study indicate that participants enjoyed discussion about topics including which games are harder, strategies for doing better at different games, and comparing scores.

We chose cognitive video gaming for the control intervention because (1) it enables non-specific attention control, (2) there is minimal concern that it will impact the primary outcome because of its non-physical nature, (3) it uses similar technology as Wii.n.Walk, (4) our feasibility data suggest that it maintains motivation and therefore decreases attrition, and (5) it is potentially beneficial and ethically acceptable [39]. Two separate trainers will administer the Wii.n.Walk and control interventions to minimize treatment bias.

### Outcome Evaluation

#### Overview

Outcomes will be evaluated (Figure 1) by blinded evaluators at baseline, end of the supervised phase (week 5), end of the unsupervised phase (week 10), and end of retention period (week 62). The evaluators will be senior university students in health sciences and will have at least 1 year of experience working with research participants. They will be trained by co-investigator, BI, who has more than 5 years of experience working with amputees and administering the outcome measures used in this study.

#### Primary Outcome Measure

The Two-Minute Walk Test (2MWT) will be used to measure walking capacity as the primary outcome measure. Starting from a standing position, participants will be asked to walk as far as they can in a safe manner for 2 minutes over an indoor, flat, out-and-back course. The distance travelled to the nearest meter is recorded. The Canadian Physical Medicine and Rehabilitation Association’s Amputee Special Interest Group [40], and others [41,42], have recommended the 2MWT as the preferred measure of walking capacity. It is used in more trials of individuals with LLA [43-53] than any other measure, enabling us to compare our results with previous studies. The 2MWT has been validated with a number of LLA samples [41,42,51-53]. The 2MWT has demonstrated intra-rater reliability (intraclass correlation coefficient/ICC=.96), inter-rater reliability (ICC=.98) [51], and validity and responsiveness to change (mean 13.6, SD 19.9 meters) in individuals with LLA [52].

#### Secondary Outcome Measures

The Short Physical Performance Battery (SPPB) will capture timed standing balance (parallel foot stance, semi-tandem, or tandem: 10 seconds each), lower limb strength captured using time (to the nearest second) taken to complete five sit-to-stand chair transfers (no hand support), and gait speed (to the nearest second) over 4 meters using a standing start [54]. There is support for test-retest reliability (ICC=.92) and validity in older adults with disability [55,56]. Due to observing a ceiling effect for this measure in our earlier pilot work, we modified the scoring of the scale by timing each of the standing balance tasks for up to 30 seconds. An additional item, timed single leg stance (up to 30 seconds for each leg), was added to evaluate single leg stance balance.

The Four Square Step Test (FSST) will be used to measure dynamic standing balance. Electric tape is used to create four squares on the floor [57]. The participant is asked to step in each square, first clockwise and then counter-clockwise, without touching the tape, as fast as possible, and with use of his or her walking aid if needed. This test is timed and faster times indicate better dynamic standing balance. Scores ≥24 seconds indicate the individual is at risk for falls [58]. FSST has shown to be reliable (ICC=.98) and valid in older adults [57].

The Physical Activity Scale for the Elderly (PASE) is a self-report measure that captures information on the frequency, duration, and intensity of various physical activities [59]. The 10-item PASE has two parts: Part 1, Leisure Time Activity, has six items about involvement in daily activities such as participating in light exercise during the past 7 days. The response options are “never”, “seldom”, “sometimes”, or “often”. Information on the type and the mean time spent engaging in the activity per day is also captured. Part 2, Household Activity, has three “yes/no” items about participation in daily activities. The last question asks about number of hours per week, as well as the amount of physical activity involved, in paid or volunteer work. The amount of time spent and participation (yes/no) are multiplied by a weighted value. The total PASE score is derived by summing each contribution and varies from 0 to 500, with higher scores representing higher physical activity levels. Test-retest reliability (ICC=.84) and validity have been reported for older adults [59].

The Activities-specific Balance Confidence (ABC) is a 16-item self-report scale to assess perceived balance confidence [60]. The item scores are summed and divided by 16 to derive a mean overall score varying from 0 to 100, with higher scores indicating more confidence. Validity and test-retest reliability (ICC=.91) have been shown in individuals with LLA [61].

#### Tertiary Outcome Measures

Life Space Assessment (LSA) is a 5-item scale that will be used to measure the size of the spatial area that an individual moves through in his or her daily life, as well as the frequency of his or her mobility within a certain timeframe [62]. Life space level (where participants travel) is measured dichotomously (yes/no), frequency is measured on a Likert Scale (1 to 4) from less than once/week to daily, and independence is measured in terms of the need for aids or equipment or assistance from another person. The total score for each item is the product of the life space level, frequency, and independence. All items are summed for a final score. Evidence for validity and test-retest reliability (*r*=.86) has been reported for older adults [63].

Modus Health Stepwatch Activity Monitor (SAM) will be mounted on the prosthetic ankle to record number of steps taken per time interval to indicate the amount of prosthetic use (Modus Health, Washington, DC). The SAM cannot be adjusted by the participant and needs to be connected to a computer with special software for programming and data downloading. It has a 99.4% accuracy in individuals with LLA for a wide range of gait styles, from slow shuffle to a fast run [64,65]. The SAM will be used to collect data in 1-week intervals at all evaluation times.

Health Utility Index Mark 3 (HUI3) is useful in performing cost-utility and cost-effectiveness analyses of new rehabilitation interventions. The HUI3 is a brief questionnaire about health status reflected in a measure of HRQOL [66]. Each single-attribute utility is scored between 0.00 and 1.00 and the multiple-attribute utility scale is scored from −0.36 to 1.00, with higher scores reflecting better health and quality of life. Test-retest reliability (ICC=.72) has been shown in patients recovering from hip fracture [67]. Differences of 0.03 have been found to represent meaningful change [68]. This study is not sufficiently powered to undertake a cost-utility analysis, but it will provide useful utility data to estimate what changes in HRQOL might be anticipated [68].

The Walking While Talking Test (WWT) is a test of divided attention to examine cognitive-motor interactions [69-72]. The WWT requires the ability to divide and switch attention between two tasks, and it has been reported that older adults show an innate preference for preserving gait over talking [73,74]. Participants walk 6 meters on a flat course, turn around, and walk 6 meters back to the start while reciting the letters of the alphabet (a, b, c, ...) aloud (WWT-simple). They repeat this routine while reciting alternate letters of the alphabet (a, c, e, …) aloud (WWT-complex). The difference in time (to the nearest second) to complete the simple and complex walks will be calculated with higher differences suggesting poorer ability to cope with dual tasks (eg, greater need to focus on walking). Inter-rater reliability (*r*=.602) and validity have been reported in older individuals [75]. The WWT will be collected with the goal of “misdirecting” participants and masking the study objectives.

The Fall Calendar will be used to document the number of falls, circumstances (eg, cause, location, assistive device used or not), and consequences participant have had (eg, medical visit, injury) over the course of study.

Adherence will be measured by total amount of the program use (minutes, frequency, and duration), which will be collected from the Wii console at the time of equipment pick-up.

### Primary Analysis

To account for any within-cluster correlation that may occur as a result of delivering the intervention in groups, ICC will be calculated among the outcomes of participants within the same groups (clusters). Variation inflation factor (VIF) will be calculated using the formula: VIF=1+ICC(M−1) [76-78]. The M variable refers to the cluster size, which equals 3 in this study. Post-treatment walking capacity scores will be compared in the Wii.n.Walk and control groups using analysis of covariance (ANCOVA) for the end of supervised phase, end of unsupervised phase, and end of retention period, controlling for site, baseline score, and possibly amputation level and age [79]. To adjust for clustering effect, the ANCOVA’s *F* statistics will be divided by the VIF [76,77]. Missing data will be handled using Multiple Imputation [80]. A sensitivity analysis will also be conducted to evaluate the impact of missing data [81]. Significance testing (*P*) and marginal means with 95% confidence intervals will be estimated. Effect size (partial eta squared) will be calculated as a ratio of the effect and total sums of squares, with a 95% confidence interval. Primary analysis will be based on intention-to-treat to include all randomized participants. However, secondary analysis on a per-protocol basis (participants who adhere to treatment) will also be conducted for comparison [82].

### Secondary/ Tertiary Analyses

ANCOVA with an adjusted *F* statistic (as explained above) will be used to compare post-treatment scores between groups for secondary and tertiary outcomes. Confidence intervals (95%) will be derived. Mean percent adherence will be calculated.

### Sample Size Calculation

The primary outcome (2MWT) was used to calculate the sample size. The responsiveness of the 2MWT in a single study that included older adults with LLA being discharged from rehabilitation had a mean of 13.6 (SD 19.9) meters [52]. In an RCT of younger (mean age 36 years) community-dwelling individuals with LLA, the mean difference between treatment and control groups was 11.2 (SD 18.4) meters [43]. Walking distance gains from our own pilot work (mean 25, SD 18.1 meters) on younger adults in rehabilitation [30], and early results from our feasibility study (mean 11.9, SD 9.3 meters) using a similar sample as the proposed study, suggests distances of up to 14 meter gains may be possible. Taking into account these data and based on our study team’s considerable clinical expertise, we decided that the minimal clinically important difference of 14 meters would be reasonable. Using the sample size calculation formula for ANCOVA in RCTs (α=.05; β=.1; rho=.72) [82], each group would require 21 participants. Borm et al [83] demonstrate that when rho lies between .2 and .8, ANCOVA further reduces the required sample size by 10-40% over change score. Accounting for an additional 4 degrees of freedom (one stratification factor for site at randomization and possibly three at analysis), an extra four participants per group (n=25) are required. Accounting for a conservative total group dropout rate of 25% and supported by physical activity clinical trials (8-24%) [84,85], 64 participants are required. Because the supervised sessions are conducted in groups of three, the sample size needs to be divisible by three. Therefore, a final n=72 will allow us to enroll a balanced n of 18 Wii.n.Walk and 18 controls at each site.

### Safety

The Wii.n.Walk manual incorporates extensive safety-related material, including teaching correct postures and avoiding unsafe situations. Any unsafe performance observed during training will be addressed immediately with corrective feedback. Participants will be instructed to regularly report their Perceived Rate of Exertion (PRE) rating from 6 to 20 (6=no exertion at all to 20=maximal exertion) to exercise in a safe zone [86]. They will also be asked to report on their pain and fatigue on a scale of 0 to 10 (0=no pain/fatigue to 10=worst possible pain/fatigue) during the session. Participants will be asked to stop the session if their PRE level is ≥14 (ie, hard or heavy PRE) or fatigue and pain levels are ≥7. For the unsupervised sessions, participants will be advised to self-monitor their own safety using the PRE and pain/fatigue scales. Participants will be encouraged to contact the site coordinator immediately if they experience any unusual discomfort, pain, or physical symptoms.

There will also be a foot/leg assessment that will be completed with the participants. At all evaluation points and at enrollment, the co-investigator, BI, or the research coordinator, will ask participants if, since their last assessment (or if at enrollment session, in the last 2 weeks), they have experienced any of the following new problems in their non-amputated and amputated limbs: skin irritation/open sores/wounds, pain, swelling, or other medical problems that have prevented/stopped them from using their prostheses as they normally would. If the participants say that they have noticed issues, BI, or in London, the research coordinator, will check the participants’ foot and stump. If BI or the research coordinator notices any issues, he or she will refer the participants for medical assessment by their medical doctor or the study doctor.

A Data and Safety Monitoring Board (DSMB) will review accumulating outcome data and advise the investigators regarding safety issues, evidence of benefit, and need for modification to the study design. The DSMB will include three members external to the research team: a statistician, a prosthetist, and an older adult with LLA. Adverse events (eg, injury, falls) will be documented by the trainer and reported to the DSMB and the applicable Ethics Review Board.

### Ethics and Funding

This study has been approved by the Ethics Boards at the University of British Columbia (Approval #: H13-01858) and Western University (Approval#: 104688), as well as the Research Review Committee for the regional health authority at each site. This study is funded by the Canadian Institutes of Health Research (CIHR) [MOP-130336], a grant from the Amputee Coalition of Canada, and the University of Alberta-Franklin Fund. BI is a Vanier Canada Graduate Scholar.

## Results

Study staff have been hired and trained at both sites and recruitment is currently underway. No participants have been enrolled yet.

## Discussion

### Overview

The lack of continuity in rehabilitation treatment after discharge leads to decreased mobility, walking capacity, functional independence, and HRQOL. Wii.n.Walk presents a promising in-home telehealth intervention that may have useful applications for older adults with LLA who are discharged from rehabilitation, live in remote areas, or have limited or no access to existing rehabilitation programs. The application of a home treatment intervention reduces the client’s burden for travel and is particularly advantageous for clients with greater disability. The benefits extend beyond improved walking capacity and include increases in physical activity tolerance and better health and participation in important life activities that may ultimately ameliorate social and financial costs.

### Limitations

This study has a number of limitations. RCTs in rehabilitation are subject to numerous threats to study validity [87]. First, blinding is a difficult limitation to overcome. Evaluators will be blinded and will request the participants not reveal their group status. We will also endeavor to mask participants to the true study objectives. It still may be evident which intervention is of primary interest based on the outcomes used. Therefore, we attempt misdirection by including the WWT test and by stating that “We are trying to determine whether cognitive or activity training is better for improving function” both in the consent form and when addressing participants’ comments/questions. Second, contamination and co-intervention will be difficult to control because the Wii Fit and the Big Brain are commercially available. While masking study objectives may reduce these risks, we will also conduct the in-clinic sessions at different times during the day so that participants will not have contact with the other group. Third, although the trainers will ask the participants not to use the treatment software outside of the treatment schedule, they may ignore these requests. Date/time stamped Wii use data downloaded at the end of intervention from the consoles will be assessed to determine adherence. This will also enable us to explore if individuals other than the participant used the Wii. Fourth, not everyone likes video games; however, the pilot work and the Wii Fit literature [72] suggest that the majority of older participants enjoy the games. Fifth, there is a possibility for a technology burden, particularly for older participants. We endeavored to simplify the technology used in this study to minimize the burden. We will use color-coded dots to highlight important buttons on the iPads, headphones, and the Wii consoles, so it will be easier for participants to locate them. Participants will be trained on how to use each piece of the technology during their in-clinic sessions as well as during the equipment set up at their homes. They will also be provided with a take-home manual that clearly explains step-by-step guidelines for using the program. In case participants have difficulty connecting with the trainer during the supervised phase, the trainer will troubleshoot remotely by telephoning participants. Sixth, our sample will represent older adult volunteers. The results will not be generalizable to younger amputees. We do not view this as a limitation given that >80% of individuals with LLA in Western countries are older adults. The results will be limited to differences related to older volunteers. Finally, loss to follow-up is a threat, particularly when participants are based remotely. To minimize loss to follow-up, the site coordinators will maintain contact with participants once a month upon termination of the unsupervised phase under the premise of collecting information on falls.

## Multimedia Appendix 1

CONSORT-EHEALTH checklist V1.6.2 [88].

## Multimedia Appendix 2

WiiNWALK 2013 CIHR peer review report.

## Abbreviations

2MWT: 2 Minute Walk Test
ABC: Activities-specific Balance Confidence
ANCOVA: analysis of covariance
CIHR: Canadian Institutes of Health Research
DSMB: Data and Safety Monitoring Board
FSST: Four Step Square Test
HRQOL: health- related quality of life
HUI3: Health Utility Index Mark 3
ICC: intraclass correlation coefficient
LLA: lower limb amputation
LSA: Life Space Assessment
PARmedX: Physical Activity Readiness Medical Examination
PAR-Q: Physical Activity Readiness Questionnaire
PASE: Physical Activity Scale for the Elderly
PRE: perceived rate of exertion
RCT: randomized controlled trial
SAM: Stepwatch Activity Monitor
SCT: Social Cognitive Theory
SPPB: Short Physical Performance Battery
SSRD: single participant research design
TF: transfemoral
TT: transtibial
VIF: variance inflation factor
WWT: Walking While Talking Test

## References

[1] Lusardi ML (2007). Orthotics and prosthetics in rehabilitation.

[2] Dillingham TR, Pezzin LE, MacKenzie EJ (2002). Limb amputation and limb deficiency: epidemiology and recent trends in the United States. South Med J.

[3] Aulivola B, Hile CN, Hamdan AD, Sheahan MG, Veraldi JR, Skillman JJ, Campbell DR, Scovell SD, LoGerfo FW, Pomposelli FB (2004). Major lower extremity amputation: outcome of a modern series. Arch Surg.

[4] Wu J, Chan TS, Bowring G (2010). Functional outcomes of major lower limb amputation 1994–2006: a modern series. J Prosthet Orthot.

[5] Andrews KL (1996). Rehabilitation in limb deficiency. 3. The geriatric amputee. Arch Phys Med Rehabil.

[6] Yiğiter K, Sener G, Erbahçeci F, Bayar K, Ulger OG, Akdoğan S (2002). A comparison of traditional prosthetic training versus proprioceptive neuromuscular facilitation resistive gait training with trans-femoral amputees. Prosthet Orthot Int.

[7] Miller WC, Speechley M, Deathe B (2001). The prevalence and risk factors of falling and fear of falling among lower extremity amputees. Arch Phys Med Rehabil.

[8] Maki BE, Holliday PJ, Topper AK (1991). Fear of falling and postural performance in the elderly. J Gerontol.

[9] Myers AM, Gonda G (2010). Research on physical activity in the elderly: practical implications for program planning. Can J Aging.

[10] van Velzen JM, van Bennekom CA, Polomski W, Slootman JR, van der Woude LH, Houdijk H (2006). Physical capacity and walking ability after lower limb amputation: a systematic review. Clin Rehabil.

[11] Jaegers SM, Arendzen JH, de Jongh HJ (1995). Prosthetic gait of unilateral transfemoral amputees: a kinematic study. Arch Phys Med Rehabil.

[12] Sansam K, Neumann V, O'Connor R, Bhakta B (2009). Predicting walking ability following lower limb amputation: a systematic review of the literature. J Rehabil Med.

[13] Davies B, Datta D (2003). Mobility outcome following unilateral lower limb amputation. Prosthet Orthot Int.

[14] Frlan-Vrgoc L, Vrbanić TS, Kraguljac D, Kovacević M (2011). Functional outcome assessment of lower limb amputees and prosthetic users with a 2-minute walk test. Coll Antropol.

[15] Munin MC, Espejo-De Guzman MC, Boninger ML, Fitzgerald SG, Penrod LE, Singh J (2001). Predictive factors for successful early prosthetic ambulation among lower-limb amputees. J Rehabil Res Dev.

[16] MacKenzie EJ, Jones AS, Bosse MJ, Castillo RC, Pollak AN, Webb LX, Swiontkowski MF, Kellam JF, Smith DG, Sanders RW, Jones AL, Starr AJ, McAndrew MP, Patterson BM, Burgess AR (2007). Health-care costs associated with amputation or reconstruction of a limb-threatening injury. J Bone Joint Surg Am.

[17] Tousignant M, Boissy P, Corriveau H, Moffet H, Cabana F (2009). In-home telerehabilitation for post-knee arthroplasty: a pilot study. Int J Telerehab.

[18] Stickland M, Jourdain T, Wong EY, Rodgers WM, Jendzjowsky NG, Macdonald GF (2011). Using telehealth technology to deliver pulmonary rehabilitation in chronic obstructive pulmonary disease patients. Can Respir J.

[19] Piotrowicz E, Korzeniowska-Kubacka I, Chrapowicka A, Wolszakiewicz J, Dobraszkiewicz-Wasilewska B, Batogowski M, Piotrowski W, Piotrowicz R (2014). Feasibility of home-based cardiac telerehabilitation: results of TeleInterMed study. Cardiol J.

[20] Tsaousides T, D'Antonio E, Varbanova V, Spielman L (2014). Delivering group treatment via videoconference to individuals with traumatic brain injury: a feasibility study. Neuropsychol Rehabil.

[21] Dishman RK (1988). Exercise adherence: its impact on public health.

[22] Jette AM, Rooks D, Lachman M, Lin TH, Levenson C, Heislein D, Giorgetti MM, Harris BA (1998). Home-based resistance training: predictors of participation and adherence. Gerontologist.

[23] Robison JI, Rogers MA (1994). Adherence to exercise programmes. Recommendations. Sports Med.

[24] Chan TC, Chan F, Shea YF, Lin OY, Luk JK, Chan FH (2012). Interactive virtual reality Wii in geriatric day hospital: a study to assess its feasibility, acceptability and efficacy. Geriatr Gerontol Int.

[25] Miller CA, Hayes DM, Dye K, Johnson C, Meyers J (2012). Using the Nintendo Wii Fit and body weight support to improve aerobic capacity, balance, gait ability, and fear of falling: two case reports. J Geriatr Phys Ther.

[26] Agmon M, Perry CK, Phelan E, Demiris G, Nguyen HQ (2011). A pilot study of Wii Fit exergames to improve balance in older adults. J Geriatr Phys Ther.

[27] Rendon AA, Lohman EB, Thorpe D, Johnson EG, Medina E, Bradley B (2012). The effect of virtual reality gaming on dynamic balance in older adults. Age Ageing.

[28] Nilsagård Ye, Forsberg AS, von Koch L (2013). Balance exercise for persons with multiple sclerosis using Wii games: a randomised, controlled multi-centre study. Mult Scler.

[29] Padala KP, Padala PR, Malloy TR, Geske JA, Dubbert PM, Dennis RA, Garner KK, Bopp MM, Burke WJ, Sullivan DH (2012). Wii-fit for improving gait and balance in an assisted living facility: a pilot study. J Aging Res.

[30] Imam B, Miller WC, McLaren L, Chapman P, Finlayson H (2013). Feasibility of the Nintendo WiiFit for improving walking in individuals with a lower limb amputation. SAGE Open Medicine.

[31] Dick JP, Guiloff RJ, Stewart A, Blackstock J, Bielawska C, Paul EA, Marsden CD (1984). Mini-mental state examination in neurological patients. J Neurol Neurosurg Psychiatry.

[32] American College of Sports Medicine (2010). ACSM's guidelines for exercise testing and prescription.

[33] Hanspal RS, Fisher K, Nieveen R (2003). Prosthetic socket fit comfort score. Disabil Rehabil.

[34] Physical Activity Readiness Questionnaire – PAR-Q.

[35] (2002). Physical Activity Readiness Medical Examination: PARmed-X.

[36] Bandura A (1997). Self-efficacy: the exercise of control.

[37] Jordan JL, Holden MA, Mason EE, Foster NE (2010). Interventions to improve adherence to exercise for chronic musculoskeletal pain in adults. Cochrane Database Syst Rev.

[38] Williams P, Lord SR (1995). Predictors of adherence to a structured exercise program for older women. Psychol Aging.

[39] Lindquist R, Wyman JF, Talley KM, Findorff MJ, Gross CR (2007). Design of control-group conditions in clinical trials of behavioral interventions. J Nurs Scholarsh.

[40] Dudek N, Deathe AB, Devlin M, Hebert J, Payne M (2010). Recommended outcome measures for lower extremity amputee rehabilitation programs. Canadian Consensus Statement, Date of Final Approval: August.

[41] Stevens P, Fross N, Kapp S (2009). Clinically relevant outcome measures in orthotics and prosthetics. The Academy Today.

[42] Deathe AB, Wolfe DL, Devlin M, Hebert JS, Miller WC, Pallaveshi L (2009). Selection of outcome measures in lower extremity amputation rehabilitation: ICF activities. Disabil Rehabil.

[43] Rau B, Bonvin F, de Bie R (2007). Short-term effect of physiotherapy rehabilitation on functional performance of lower limb amputees. Prosthet Orthot Int.

[44] Meikle B, Boulias C, Pauley T, Devlin M (2003). Does increased prosthetic weight affect gait speed and patient preference in dysvascular transfemoral amputees?. Arch Phys Med Rehabil.

[45] Resnik L, Borgia M (2011). Reliability of outcome measures for people with lower-limb amputations: distinguishing true change from statistical error. Phys Ther.

[46] Gremeaux V, Damak S, Troisgros O, Feki A, Laroche D, Perennou D, Benaim C, Casillas JM (2012). Selecting a test for the clinical assessment of balance and walking capacity at the definitive fitting state after unilateral amputation: a comparative study. Prosthet Orthot Int.

[47] Parker K, Kirby RL, Adderson J, Thompson K (2010). Ambulation of people with lower-limb amputations: relationship between capacity and performance measures. Arch Phys Med Rehabil.

[48] Bhangu S, Devlin M, Pauley T (2009). Outcomes of individuals with transfemoral and contralateral transtibial amputation due to dysvascular etiologies. Prosthet Orthot Int.

[49] Deathe AB, Miller WC (2005). The L test of functional mobility: measurement properties of a modified version of the timed "up & go" test designed for people with lower-limb amputations. Phys Ther.

[50] Devlin M, Pauley T, Head K, Garfinkel S (2004). Houghton Scale of prosthetic use in people with lower-extremity amputations: Reliability, validity, and responsiveness to change. Arch Phys Med Rehabil.

[51] Brooks D, Hunter JP, Parsons J, Livsey E, Quirt J, Devlin M (2002). Reliability of the two-minute walk test in individuals with transtibial amputation. Arch Phys Med Rehabil.

[52] Brooks D, Parsons J, Hunter JP, Devlin M, Walker J (2001). The 2-minute walk test as a measure of functional improvement in persons with lower limb amputation. Arch Phys Med Rehabil.

[53] Miller WC, Deathe AB, Speechley M (2001). Lower extremity prosthetic mobility: a comparison of 3 self-report scales. Arch Phys Med Rehabil.

[54] Guralnik JM, Simonsick EM, Ferrucci L, Glynn RJ, Berkman LF, Blazer DG, Scherr PA, Wallace RB (1994). A short physical performance battery assessing lower extremity function: association with self-reported disability and prediction of mortality and nursing home admission. J Gerontol.

[55] Ostir GV, Volpato S, Fried LP, Chaves P, Guralnik JM, Women's Health and Aging Study (2002). Reliability and sensitivity to change assessed for a summary measure of lower body function: results from the Women's Health and Aging Study. J Clin Epidemiol.

[56] Freiberger E, de Vreede P, Schoene D, Rydwik E, Mueller V, Frändin K, Hopman-Rock M (2012). Performance-based physical function in older community-dwelling persons: a systematic review of instruments. Age Ageing.

[57] Dite W, Temple VA (2002). A clinical test of stepping and change of direction to identify multiple falling older adults. Arch Phys Med Rehabil.

[58] Dite W, Connor HJ, Curtis HC (2007). Clinical identification of multiple fall risk early after unilateral transtibial amputation. Arch Phys Med Rehabil.

[59] Washburn RA, McAuley E, Katula J, Mihalko SL, Boileau RA (1999). The Physical Activity Scale for the Elderly (PASE): evidence for validity. J Clin Epidemiol.

[60] Powell LE, Myers AM (1995). The Activities-specific Balance Confidence (ABC) Scale. J Gerontol A Biol Sci Med Sci.

[61] Miller WC, Deathe AB, Speechley M (2003). Psychometric properties of the Activities-specific Balance Confidence Scale among individuals with a lower-limb amputation. Arch Phys Med Rehabil.

[62] Stalvey BT, Owsley C, Sloane ME, Ball K (1999). The Life Space Questionnaire: A measure of the extent of mobility of older adults. Journal of Applied Gerontology.

[63] Baker PS, Bodner EV, Allman RM (2003). Measuring life-space mobility in community-dwelling older adults. J Am Geriatr Soc.

[64] Modus Health (2014). Stepwatch.

[65] Coleman KL, Boone DA, Smith DG, Mathews DE, Laing LS (1998). Quantification of prosthetic outcome: Flex Walk-II vs. Sach foot.

[66] Torrance GW, Furlong W, Feeny D (2002). Health utility estimation. Expert Rev Pharmacoecon Outcomes Res.

[67] Jones CA, Feeny D, Eng K (2005). Test-retest reliability of health utilities index scores: evidence from hip fracture. Int J Technol Assess Health Care.

[68] Drummond M (2001). Introducing economic and quality of life measurements into clinical studies. Ann Med.

[69] Sheridan PL, Solomont J, Kowall N, Hausdorff JM (2003). Influence of executive function on locomotor function: divided attention increases gait variability in Alzheimer's disease. J Am Geriatr Soc.

[70] Camicioli R, Howieson D, Lehman S, Kaye J (1997). Talking while walking: the effect of a dual task in aging and Alzheimer's disease. Neurology.

[71] Bootsma-van der Wiel A, Gussekloo J, de Craen AJ, van Exel E, Bloem BR, Westendorp RG (2003). Walking and talking as predictors of falls in the general population: the Leiden 85-Plus Study. J Am Geriatr Soc.

[72] de Hoon EW, Allum JH, Carpenter MG, Salis C, Bloem BR, Conzelmann M, Bischoff HA (2003). Quantitative assessment of the stops walking while talking test in the elderly. Arch Phys Med Rehabil.

[73] Woollacott M, Shumway-Cook A (2002). Attention and the control of posture and gait: a review of an emerging area of research. Gait Posture.

[74] Schrodt LA, Mercer VS, Giuliani CA, Hartman M (2004). Characteristics of stepping over an obstacle in community dwelling older adults under dual-task conditions. Gait Posture.

[75] Verghese J, Buschke H, Viola L, Katz M, Hall C, Kuslansky G, Lipton R (2002). Validity of divided attention tasks in predicting falls in older individuals: a preliminary study. J Am Geriatr Soc.

[76] Klar N, Donner A (2001). Current and future challenges in the design and analysis of cluster randomization trials. Stat Med.

[77] Campbell MK, Mollison J, Steen N, Grimshaw JM, Eccles M (2000). Analysis of cluster randomized trials in primary care: a practical approach. Fam Pract.

[78] Murray DM, Blistein JL (2003). Methods to reduce the impact of intraclass correlation in group-randomized trials. Eval Rev.

[79] Van Breukelen GJ (2006). ANCOVA versus change from baseline: more power in randomized studies, more bias in nonrandomized studies [corrected]. J Clin Epidemiol.

[80] Streiner DL (2002). The case of the missing data: methods of dealing with dropouts and other research vagaries. Can J Psychiatry.

[81] Fielding S, Maclennan G, Cook JA, Ramsay CR (2008). A review of RCTs in four medical journals to assess the use of imputation to overcome missing data in quality of life outcomes. Trials.

[82] Moncur R, Larmer J (2009). Clinical applicability of intention-to-treat analyses. Evidence Based Medicine.

[83] Borm GF, Fransen J, Lemmens WA (2007). A simple sample size formula for analysis of covariance in randomized clinical trials. J Clin Epidemiol.

[84] Blanton S, Morris DM, Prettyman MG, McCulloch K, Redmond S, Light KE, Wolf SL (2006). Lessons learned in participant recruitment and retention: the EXCITE trial. Phys Ther.

[85] Duncan P, Studenski S, Richards L, Gollub S, Lai SM, Reker D, Perera S, Yates J, Koch V, Rigler S, Johnson D (2003). Randomized clinical trial of therapeutic exercise in subacute stroke. Stroke.

[86] Borg GAV (1998). Borg's Perceived exertion and pain scales.

[87] Andrews K (1991). The limitations of randomized controlled trials in rehabilitation research. Clinical Rehabilitation.

[88] Eysenbach G, CONSORT-EHEALTH Group (2011). CONSORT-EHEALTH: improving and standardizing evaluation reports of Web-based and mobile health interventions. J Med Internet Res.

