# Supplementary material for: A Telehealth Intervention Using Nintendo Wii Fit Balance Boards and iPads to Improve Walking in Older Adults With Lower Limb Amputation (Wii.n.Walk): Study Protocol for a Randomized Controlled Trial
Source: JMIR Res Protoc. 2014 Dec 22;3(4):e80. doi: 10.2196/resprot.4031 (PMC4376145; doi:10.2196/resprot.4031)
Supplement: Supplementary file 1 [file resprot_v3i4e80_app1.pdf]

CONSORT-EHEALTH Checklist V1.6.2 Report

(based on CONSORT-EHEALTH V1.6), available at [http://tinyurl.com/consort-ehealth-v1-6].

4031

Date completed

11/14/2014 16:24:48

by

Bita Imam

The phrase "A Tablet-enabled Teleheatlh Intervention" is used in the title. We do not highlight our use of the Nintendo Wii Fit Gaming Software (virtual reality computer-based games) in the title due to length constraints.

TITLE

1a-i) Identify the mode of delivery in the title

The phrase "A Tablet-enabled Teleheatlh Intervention" is used in the title. We do not highlight our use of the Nintendo Wii Fit Gaming Software (virtual reality computer-based games) in the title due to length constraints.

1a-ii) Non-web-based components or important co-interventions in title

1a-iii) Primary condition or target group in the title

"Older Adults with Lower Limb Amputation"

ABSTRACT

1b-i) Key features/functionalities/components of the intervention and comparator in the METHODS section of the ABSTRACT

"Participants will be stratified by site and block randomized in triplets to either the Wii.n.Walk intervention or an attention control group employing the Wii Big Brain cognitive software. This trial will include both supervised and unsupervised phases. During the supervised phase, both groups will receive 40-minute sessions of supervised group training three times per week for a duration of four weeks. Participants will complete the first week of the intervention in groups of three at their local rehabilitation center with a trainer. The remaining three weeks will take place at participants' homes using remote supervision by the trainer using Apple iPad technology. At the end of four weeks, the supervised period will end and the unsupervised period will begin. Participants will retain the Wii console and encouraged to continue using the program for an additional four weeks duration."

1b-ii) Level of human involvement in the METHODS section of the ABSTRACT

1b-iii) Open vs. closed, web-based (self-assessment) vs. face-to-face assessments in the METHODS section of the ABSTRACT

1b-iv) RESULTS section in abstract must contain use data

1b-v) CONCLUSIONS/DISCUSSION in abstract for negative trials

INTRODUCTION

2a-i) Problem and the type of system/solution

"In 2003, it was estimated that more than two million individuals were living with lower limb amputation (LLA) in North America with an annual incidence of 150,000 [1]. Over half of LLAs are transtibial (TT) and transfemoral (TF) amputations [2]. [...] Recovery following LLA is notably slow. A lengthy recovery process is especially common among older adults who often have multiple co-morbidities including peripheral vascular disease, peripheral neuropathy, hypertension, heart disease [3,4], and cognitive impairment [5]. LLA, compounded with these co-morbidities, influences walking and places these individuals at a high risk of falling and sustaining injury after a fall [6]. In fact, 52% of community-dwelling individuals with LLA report falling each year [7]. Similarly, 49% have a fear of falling and 65% report low balance confidence [7]. The consequences associated with these numbers may contribute to deterioration in balance [8], endurance, strength, and coordination [9] in older adults and ultimately a decline in walking capacity. [...]

Following a LLA, individuals need to participate in prosthetic rehabilitation. Rehabilitation includes procurement of a prosthetic limb and ambulation training. The costs associated with post-amputation care and prosthetic rehabilitation are considerable. [...] Therefore, accessible and innovative approaches are needed to improve outcomes for individuals with LLA and overcome these barriers to participation.

In-home telehealth is an innovative and emerging approach to provide rehabilitation through technologies and telecommunication [17-20]. Home treatments create accessible rehabilitation programs and promote continuity of care after discharge; they also offset the time and expense of travel for clients to in-hospital rehabilitation programs [21,22]. Access to in-home rehabilitation is particularly important for those with limited access to facilities and transportation [23]."

"Nintendo Wii FitTM is a commercial gaming technology that shows promise as an in-home rehabilitation tool."

2a-ii) Scientific background, rationale: What is known about the (type of) system

"The benefits of using Wii FitTM technology as a rehabilitation tool is demonstrated in the growing knowledge base on the use of gaming technology in older adult rehabilitation. In a study of older adults that used Wii FitTM during in-patient rehabilitation, more than 80% expressed their desire to continue using Wii FitTM at home [24]. Preliminary evidence suggests Wii FitTM training is a feasible and safe method leading to improvements in balance [25,26], walking [25-27] and balance confidence in older adults [27]. Studies have reported improvements in walking and balance confidence in individuals with multiple sclerosis [28] and improved balance and decreased risk of falls in individuals with mild Alzheimer's [29]. Pilot testing has shown improvements in balance, balance confidence, and gait variables [25] in two older adults with LLA, which is consistent with findings from our own pilot work [30].

In a Single Subject Research Design (SSRD) study of six individuals with LLA, the feasibility of a Wii FitTM oriented intervention consisting of structured daily training varying from two to six weeks was assessed. Results indicated a statistical improvement in walking capacity in five participants who had three or greater than three weeks of intervention [30].

[…] The aim of this study is to provide a useful foundation for which to extend further study on this topic and conduct a randomized controlled trial (RCT) to assess our in-home telehealth Wii FitTM intervention protocol we call Wii.n.Walk."

The comparator was chosen because it uses the same gaming system as the Wii FitTM and provides a non-specific attention control.

METHODS

3a) CONSORT: Description of trial design (such as parallel, factorial) including allocation ratio

"The primary clinical hypothesis is that participants in the Wii.n.Walk intervention group will experience an improvement in walking capacity compared to the control group. The secondary clinical hypothesis is that participants in the Wii.n.Walk intervention group will experience an improvement in lower limb functioning (balance, gait speed, and strength), dynamic balance, physical activity, and balance confidence. The tertiary clinical hypothesis suggests that the Wii.n.Walk group will experience an improvement in life space mobility, prosthetic use, HRQOL, and will have a lower incidence of falls. The adherence hypothesis is that the Wii.n.Walk group will have ≥ 80% adherence."

3b) CONSORT: Important changes to methods after trial commencement (such as eligibility criteria), with reasons

This item is not relevant because the manuscript is a protocol (no changes have been made to the methods).

3b-i) Bug fixes, Downtimes, Content Changes

4a) CONSORT: Eligibility criteria for participants

"Participants need to: be ≥ 50 years of age, have a unilateral TT or TF amputation, use their prosthesis for at least two hours per day for the past six months to minimize the influence of residual limb/prosthetic fit problems, be cognitively able to engage in the program (receive a score on the Modified Mini-mental Status Exam score of > 23) [31], and have a television that will enable connection to the Nintendo hardware.

Individuals will be excluded if they: cannot communicate in English, cannot provide informed consent, have medical conditions (e.g., congestive heart failure) that limit exercise participation as determined using the American College of Sports Medicine exercise guidelines for older adults [32], have prosthetic fit issues (e.g., pain and discomfort) as indicated by scores < 6 on the Prosthetic Socket Fit Comfort Scale [33], or are currently participating in another supervised exercise or training program (e.g., balance training)."

4a-i) Computer / Internet literacy

4a-ii) Open vs. closed, web-based vs. face-to-face assessments:

“A total of 72 community-dwelling prosthetic ambulators in London, Ontario and Vancouver, British Columbia will be recruited through clinicians and prosthetists. A letter of information will be distributed to all individuals in the amputee program databases who meet the study inclusion criteria.

Participants will complete two training phases: a 4-week Supervised Phase (3 times/week, 40 minutes training /session) followed by a 4-week Unsupervised Phase.[...] Participants will meet in groups of three at their local rehabilitation centre with an experienced trainer during the first week to learn the program. They will then complete the final three weeks of the Supervised program at home while being remotely supervised by the trainer and remotely interacting with the other two group participants. During the Supervised Phase, the trainer will provide individualized intervention and advance the training as the participant improves. At the end of the 4-week Supervised Phase, participants will retain the Wii units and be encouraged to use the program on their own for an additional 4 weeks (Unsupervised Phase).

[...] Supervised Phase home sessions will be monitored by a trainer remotely using iPads with wifi + cellular (Apple Inc., Cupertino, California, USA) preloaded with the VidyoMobile videoconferencing application (Vidyo Inc., Hackensack, New Jersey, USA). VidyoMobile enables the participant to meet at home with the trainer and the other two participants in the group.
[...]

Control Intervention
The control group will follow the same protocol but will be trained to use the Wii Big Brain AcademyTM Degree program (Nintendo, Kyoto, Japan)."

4a-iii) Information giving during recruitment

4b) CONSORT: Settings and locations where the data were collected

"Vancouver, BC and London, ON"

4b-i) Report if outcomes were (self-)assessed through online questionnaires

This RCT does not use online questionnaires.

4b-ii) Report how institutional affiliations are displayed

5) CONSORT: Describe the interventions for each group with sufficient details to allow replication, including how and when they were actually administered

5-i) Mention names, credential, affiliations of the developers, sponsors, and owners

5-ii) Describe the history/development process

5-iii) Revisions and updating

5-iv) Quality assurance methods

5-v) Ensure replicability by publishing the source code, and/or providing screenshots/screen-capture video, and/or providing flowcharts of the algorithms used

5-vi) Digital preservation

5-vii) Access

"Supervised Phase home sessions will be monitored by a trainer remotely using iPads with wifi + cellular (Apple Inc., Cupertino, California, USA) preloaded with the VidyoMobile videoconferencing application (Vidyo Inc., Hackensack, New Jersey, USA). VidyoMobile enables the participant to meet at home with the trainer and the other two participants in the group. For better sound quality, participants will be asked to wear wireless headphones (KinivoTM, Bellevue, Washington USA) with noise cancellation features. The iPad interface is simplified as much as possible. Only the VidyoMobile app is available to the participants (access to all other apps is disabled through the iPad's parental control feature), and they can connect to the trainer by entering only their names and a simple PIN code. iPads will be securely mounted on a sturdy tablet tripod and will be placed a few meters away from participant's TV and behind the participant, so that the trainer can see both the participant's screen as well as his or her posture. The ideal location will be established by the trainer during the home setup of the Wii.n.Walk equipment. At the beginning of each session, the trainer will hold a brief discussion session with all participants, through the videoconferencing software, to review the plan for the session and address any questions. Once the session begins, the trainer is able to watch and supervise all three participants from his or her desktop/laptop at the clinic. The participant's iPad can display the trainer and the other two participants. The trainer can remotely deactivate each iPad's camera to reduce distractions while exercising or to reduce video streaming costs. The trainer will activate the iPad's camera on at least two occasions during each session to enable opportunities for vicarious learning and participant-to-participant verbal persuasion. As an example, the trainer will ask two participants to watch the third perform an exercise. Verbal persuasion will be provided by the trainer to the participant being watched. [...]

At the end of the Supervised Phase, the iPads, stands, and headphones will be collected by the trainer, while participants will retain the Wii console and balance board for the Unsupervised Phase."

5-viii) Mode of delivery, features/functionalities/components of the intervention and comparator, and the theoretical framework

"Participants in the experimental group will receive the Wii.n.Walk intervention. The Wii.n.Walk intervention was developed by core members of the research team and refined based on observations and the feedback received from the participants in the pilot studies [30]. Modifications for trainer instructions were made to Wii FitTM postures and activities to prevent incorrect postures/techniques and to promote function and safety. Preliminary work also informed the dosage/frequency and duration of the intervention.

Social Cognitive Theory [36] (SCT) is the theoretical foundation to the Wii.n.Walk intervention. This theory was developed to enhance all four sources of self-efficacy: performance mastery, vicarious learning, verbal persuasion, and reinterpretation of physiological responses. Performance mastery, or learning to perform a specific skill, is the most robust source of self-efficacy. Successful performance of the Wii.n.Walk activities may provide a sense of accomplishment and thereby improve self-efficacy. Vicarious learning, or learning by watching others successfully accomplish activities, provides the observer a sense that they, too, have the ability to accomplish the task. This will be established by performing the Wii.n.Walk activities in groups initially and having participants watch the other group members perform the activities. Verbal persuasion arises from credible feedback, guiding the learner through the task, and motivating his or her best effort. The trainer will provide this feedback when appropriate; at least once each session for each group member. The Nintendo device also automatically provides auditory and visual feedback based on the participant's performance. Finally, participants will be taught to reinterpret physiological responses (e.g., stress and anxiety) that may be associated with challenging Wii.n.Walk activities. According to a systematic review, physical activity programs that incorporate SCT are more effective in enhancing adherence [37]. More specifically, social support, peer modeling, and group training have been identified as important factors for increasing adherence in older adults [38].

The Wii.n.Walk protocol consists of Wii FitTM activities. Participants stand on the Wii FitTM balance board and play the games through weight shifting or by using the Wii remote control. The intervention protocol includes selected activities and exercises including yoga (static and dynamic single and double leg poses), balance tasks (lateral, posterior and anterior weight shifting exercises), strength training (dynamic single and double leg exercises), and aerobics (running on the spot and step class). [...]

The activities will be chosen by the trainer based on the participants' abilities. By default, the more challenging levels of the games are initially locked and can only be unlocked if the participant successfully completes easier, prerequisite levels. In addition, progression to more difficult and longer activities is guided by instructions in the Wii.n.Walk manual. The manual also provides modified activity positions such as adding unilateral or bilateral external hand support if required by the participant. Modifications can be made if the participant has difficulty or is unable to do the activity. As an example, activities may be modified for an individual with a TF amputation if the prosthesis is not structurally capable of assuming the exercise position (e.g., some of the exercises require stance phase prosthetic knee flexion). Common postural mistakes are included in the manual to guide the trainer in correcting positioning. [...]

The control group will follow the same protocol but will be trained to use the Wii Big Brain AcademyTM Degree program (Nintendo, Kyoto, Japan). Big Brain is a low-cost, commercially available, software consisting of video games to improve cognitive function. Participants will use the Wii remote to participate in the games by pointing and clicking to select answers in response to on-screen questions. Big Brain games require participants to identify, memorize, analyze, compute, and visualize. The games have easy, medium, and difficult levels. Participants initially start with easy games and progress to more challenging levels based on their performance. The trainer will design the Supervised sessions, provide instruction/feedback, and facilitate group discussions. Results from the feasibility study indicate that participants enjoyed discussion about topics including which games are harder, strategies for doing better at different games, and comparing scores.

We chose cognitive video gaming for the control intervention because: 1) it enables non-specific attention control, 2) there is minimal concern that it will impact the primary outcome because of its non-physical nature, 3) it uses similar technology as Wii.n.Walk, 4) our feasibility data suggest that it maintains motivation and therefore decreases attrition, and 5) it is potentially beneficial and ethically acceptable [39]. Two separate trainers will administer the Wii.n.Walk and control interventions to minimize treatment bias."

5-ix) Describe use parameters

5-x) Clarify the level of human involvement

5-xi) Report any prompts/reminders used

There are no prompts/reminders necessary for this study.

5-xii) Describe any co-interventions (incl. training/support)

"Participants will meet in groups of three at their local rehabilitation centre with an experienced trainer during the first week to learn the program."

6a) CONSORT: Completely defined pre-specified primary and secondary outcome measures, including how and when they were assessed

"Outcomes will be evaluated (Figure 1) by a blinded evaluator at baseline, end of Supervised Phase (week 5), end of Unsupervised Phase (week 10), and end of retention period (week 62).

a. Primary outcome measure.
1. The 2 Minute Walk Test (2MWT) will be used to measure walking capacity as the primary outcome measure. Starting from a standing position, participants will be asked to walk as far as they can in a safe manner for two minutes over an indoor flat out course. The distance travelled to the nearest metre is recorded. The Canadian Physical Medicine and Rehabilitation Association's Amputee Special Interest Group [40] and others [41,42] have recommended the 2MWT as the preferred measure of walking capacity. It is used in more trials of individuals with LLA43-53 than any other measure, enabling us to compare our results with previous studies. The 2MWT has been validated with a number of LLA samples [41,42,51-53]. The 2MWT has demonstrated intra-rater reliability (ICC=0.96), inter-rater reliability (ICC=0.98) [51], and validity and responsiveness to change (mean=13.6; SD=19.9 metres) in individuals with LLA [52].

b. Secondary outcome measures.
2. The Short Physical Performance Battery (SPPB) will capture timed standing balance (parallel foot stance, semi-tandem, or tandem: 10 seconds each), lower limb strength captured using time (to the nearest second) taken to complete five sit to stand chair transfers (no hand support), and gait speed (to the nearest second) over four metres using a standing start [54]. There is support for test-retest reliability (ICC=0.92) and validity in older adults with disability [55,56]. Due to observing a ceiling effect for this measure in our earlier pilot work, we modified the scoring of the scale by timing each of the standing balance tasks for up to 30 seconds. An additional item was also added: timed single leg stance (up to 30 seconds for each leg) to evaluate single leg stance balance.
3. The Four Square Step Test (FSST) will be used to measure dynamic standing balance. Electric tape is used to create four squares on the floor [57]. The participant is asked to step in each square, first clockwise and then counter-clockwise, without touching the tape as fast as possible and with use of his or her walking aid if needed. This test is timed and faster times indicate better dynamic standing balance. Scores ≥ 24 seconds indicate the individual is at risk for falls [58]. FSST has shown to be reliable (ICC=0.98) and valid in older adults [57].
4. The Physical Activity Scale for the Elderly (PASE) is a self-report measure that captures information on the frequency, duration, and intensity of various physical activities [59]. The 10-item PASE has two parts: part one, Leisure Time Activity, has six items about involvement in daily activities such as participating in light exercise during the past seven days. The response options are "never", "seldom", "sometimes", or "often". Information on the type and the mean time spent engaging in the activity per day is also captured. Part two, Household Activity, has three yes/no items about participation in daily activities. The last question asks about number of hours per week, as well as the amount of physical activity involved in paid or volunteer work. The amount of time spent and participation (yes/no) are multiplied by a weighted value. The total PASE score is derived by summing each contribution and varies from 0 to 500, with higher scores representing higher physical activity levels. Test-retest reliability (ICC=0.84) and validity have been reported for older adults [59].
5. The Activities-specific Balance Confidence (ABC) is a 16-item self-report scale to assess perceived balance confidence [60]. The item scores are summed and divided by 16 to derive a mean overall score varying from 0 to 100, with higher scores indicating more confidence. Validity and test-retest reliability (ICC=0.91) have been shown in individuals with LLA [61].

c. Tertiary outcome measures.
6. Life Space Assessment (LSA) is a 5-item scale that will be used to measure the size of the spatial area that an individual moves through in his or her daily life, as well as the frequency of his or her mobility within a certain timeframe [62]. Life space level (where participants travel) is measured dichotomously (yes/no), frequency is measured on a Likert Scale (1 to 4) from less than once/week to daily, and independence is measured in terms of the need for aids or equipment or assistance from another person. The total score for each item is the product of the life space level, frequency, and independence. All items are summed for a final score. Evidence for validity and test-retest reliability (r=0.86) has been reported for older adults [63].
7. Modus Health StepwatchTM Activity Monitor (SAM) will be mounted on the prosthetic ankle to record number of steps taken per time interval to indicate the amount of prosthetic use (Modus Health, Washington, DC). The SAM cannot be adjusted by the participant and needs to be connected to a computer with special software for programming and data downloading. It has a 99.4% accuracy in individuals with LLA for a wide range of gait styles, from slow shuffle to a fast run [64,65]. The SAM will be used to collect data in 1-week intervals at all evaluation times.
8. Health Utility Index Mark 3 (HUI3) is useful in performing cost-utility and cost-effectiveness analyses of new rehabilitation interventions. The HUI3 is a brief questionnaire about health status reflected in a measure of health-related quality of life (HRQOL) [66]. Each single-attribute utility is scored between 0.00 and 1.00 and the multiple-attribute utility scale is scored from -0.36 to 1.00, with higher scores reflecting better health and quality of life. Test-retest reliability (ICC = 0.72) has been shown in patients recovering from hip fracture [67]. Differences of 0.03 have been found to represent meaningful change [68]. This study is not sufficiently powered to undertake a cost-utility analysis, but it will provide useful utility data to estimate what changes in HRQOL might be anticipated [68].
9. The Walking While Talking Test (WWT) is a test of divided attention to examine cognitive-motor interactions [69-72]. The WWT requires the ability to divide and switch attention between two tasks, and it has been reported that older adults show an innate preference for preserving gait over talking [73,74]. Participants walk six meters on a flat course, turn around, and walk six meters back to the start while reciting the letters of the alphabet (a, b, c, ...) aloud (WWT-simple). They repeat this routine while reciting alternate letters of the alphabet (a, c, e, …) aloud (WWT-complex). The difference in time (to the nearest second) to complete the simple and complex walks will be calculated with higher differences suggesting poorer ability to cope with dual tasks (e.g., greater need to focus on walking). Inter-rater reliability (r=0.602) and validity have been reported in older individuals [75]. The WWT will be collected with the goal of 'misdirecting' participants and masking the study objectives.
10. The Fall Calendar will be used to document the number of falls, circumstances (e.g., cause, location, assistive device used or not), and consequences participant have had (e.g., medical visit, injury) over the course of study.
11. Adherence: Total amount of the program use (minutes, frequency, and duration) will be collected from the Wii console at the time of equipment pick up."

6a-i) Online questionnaires: describe if they were validated for online use and apply CHERRIES items to describe how the questionnaires were designed/deployed

6a-ii) Describe whether and how "use" (including intensity of use/dosage) was defined/measured/monitored

6a-iii) Describe whether, how, and when qualitative feedback from participants was obtained

6b) CONSORT: Any changes to trial outcomes after the trial commenced, with reasons
The manuscript is a protocol, thus this item is not applicable.
7a) CONSORT: How sample size was determined
7a-i) Describe whether and how expected attrition was taken into account when calculating the sample size

7b) CONSORT: When applicable, explanation of any interim analyses and stopping guidelines
The manuscript is a protocol, thus this item is not applicable.
8a) CONSORT: Method used to generate the random allocation sequence

“To ensure balance between groups and masking of group assignment, a central computerized randomization process will be designed by the research team statistician, with undisclosed variable block sizes.”

8b) CONSORT: Type of randomisation; details of any restriction (such as blocking and block size)

“Participants will be stratified by site and block randomized to the Wii.n.Walk intervention or control group in triplets using a 1:1 allocation ratio.”

9) CONSORT: Mechanism used to implement the random allocation sequence (such as sequentially numbered containers), describing any steps taken to conceal the sequence until interventions were assigned

“Randomization will occur after the participant is screened and enrolled (Figure 1).”

10) CONSORT: Who generated the random allocation sequence, who enrolled participants, and who assigned participants to interventions

“The site coordinators will contact the statistician via telephone or email and obtain group assignment. The participant’s contact information will be forwarded to the appropriate group trainer to arrange for an initial training session.”

11a) CONSORT: Blinding - If done, who was blinded after assignment to interventions (for example, participants, care providers, those assessing outcomes) and how

11a-i) Specify who was blinded, and who wasn’t

“Evaluators will be blinded and will request the participants not reveal their group status. We will also endeavor to mask participants to the true study objectives. It still may be evident which intervention is of primary interest based on the outcomes used. Therefore we attempt misdirection by including the WWT test and by stating that “We are trying to determine whether cognitive or activity training is better for improving function” both in the consent form and when addressing participants’ comments/questions.”

11a-ii) Discuss e.g., whether participants knew which intervention was the “intervention of interest” and which one was the “comparator”

11b) CONSORT: If relevant, description of the similarity of interventions

“We chose cognitive video gaming for the control intervention because: […] 3) it uses similar technology as Wii.n.Walk […]”

12a) CONSORT: Statistical methods used to compare groups for primary and secondary outcomes

“Primary Analysis

[…] Post-treatment walking capacity scores will be compared in the Wii.n.Walk and control groups using analysis of covariance (ANCOVA) for the end of Supervised Phase, end of Unsupervised Phase, and end of retention period, controlling for site, baseline score, and possibly amputation level and age [79]. To adjust for clustering effect, the ANCOVA’s F statistics will be divided by the VIF [76,77]. […] Significance testing (p) and marginal means with 95% confidence intervals will be estimated. Effect size (partial 2) will be calculated as a ratio of the effect and total sums of squares, with a 95% confidence interval. Primary analysis will be based on intention-to-treat to include all randomized participants. However, secondary analysis on a per-protocol basis (participants who adhere to treatment) will also be conducted for comparison [82].

Secondary/ Tertiary Analyses

ANCOVA with an adjusted F statistic (as explained above) will be used to compare post-treatment scores between groups for secondary and tertiary outcomes. Confidence intervals (95%) will be derived. Mean percent adherence will be calculated.”

12a-i) Imputation techniques to deal with attrition / missing values

“Missing data will be handled using Multiple Imputation [80]. A sensitivity analysis will also be conducted to evaluate the impact of missing data [81].”

12b) CONSORT: Methods for additional analyses, such as subgroup analyses and adjusted analyses

“To account for any within-cluster correlation that may occur as a result of delivering the intervention in groups, intraclass correlation coefficient (ICC) will be calculated among the outcomes of participants within the same groups (clusters). Variation inflation factor (VIF) will be calculated using the formula: VIF = 1 + ICC (M - 1) [76-78]. The M variable refers to the cluster size which equals three in this study.”

RESULTS

13a) CONSORT: For each group, the numbers of participants who were randomly assigned, received intended treatment, and were analysed for the primary outcome

This item is not relevant to the manuscript, because it is a protocol.

13b) CONSORT: For each group, losses and exclusions after randomisation, together with reasons

This item is not relevant to the manuscript, because it is a protocol.

13b-i) Attrition diagram

14a) CONSORT: Dates defining the periods of recruitment and follow-up

This item is not relevant to the manuscript, because it is a protocol.

14a-i) Indicate if critical “secular events” fell into the study period

14b) CONSORT: Why the trial ended or was stopped (early)

This item is not relevant to the manuscript, because it is a protocol.

15) CONSORT: A table showing baseline demographic and clinical characteristics for each group

This item is not relevant to the manuscript, because it is a protocol.

15-i) Report demographics associated with digital divide issues

This item is not relevant to the manuscript, because it is a protocol.

16a) CONSORT: For each group, number of participants (denominator) included in each analysis and whether the analysis was by original assigned groups

16-i) Report multiple “denominators” and provide definitions

This item is not relevant to the manuscript, because it is a protocol.

16-ii) Primary analysis should be intent-to-treat

17a) CONSORT: For each primary and secondary outcome, results for each group, and the estimated effect size and its precision (such as 95% confidence interval)

This item is not relevant to the manuscript, because it is a protocol.

17a-i) Presentation of process outcomes such as metrics of use and intensity of use

17b) CONSORT: For binary outcomes, presentation of both absolute and relative effect sizes is recommended

This item is not relevant to the manuscript, because it is a protocol.

18) CONSORT: Results of any other analyses performed, including subgroup analyses and adjusted analyses, distinguishing pre-specified from exploratory

This item is not relevant to the manuscript, because it is a protocol.

18-i) Subgroup analysis of comparing only users

19) CONSORT: All important harms or unintended effects in each group

This item is not relevant to the manuscript, because it is a protocol.

19-i) Include privacy breaches, technical problems

19-ii) Include qualitative feedback from participants or observations from staff/researchers

DISCUSSION

20) CONSORT: Trial limitations, addressing sources of potential bias, imprecision, multiplicity of analyses

20-i) Typical limitations in ehealth trials

“This study has a number of limitations. RCTs in rehabilitation are subject to numerous threats to study validity [87]. 1) Blinding is a difficult limitation to overcome. Evaluators will be blinded and will request the participants not reveal their group status. We will also endeavor to mask participants to the true study objectives. It still may be evident which intervention is of primary interest based on the outcomes used. Therefore we attempt misdirection by including the WWT test and by stating that “We are trying to determine whether cognitive or activity training is better for improving function” both in the consent form and when addressing participants’ comments/questions. 2) Contamination and co-intervention will be difficult to control because the Wii FitTM and the Big Brain are commercially available. While masking study objectives may reduce these risks, we will also conduct the in-clinic sessions at different times during the day so that participants will not have contact with the other group. 3) Although the trainers will ask the participants not to use the treatment software outside of the treatment schedule, they may ignore these requests. Date/time stamped Wii use data downloaded at the end of intervention from the consoles will be assessed to determine adherence. This will also enable us to explore if individuals other than the participant used the Wii. 4) Not everyone likes video games. However, the pilot work and the Wii FitTM literature [72] suggest that the majority of older participants enjoy the games. 5) There is a possibility for a technology burden, particularly for older participants. We endeavoured to simplify the technology used in this study to minimize the burden. We will use color-coded dots to highlight important buttons on the iPads, headphones, and the Wii consoles so it will be easier for participants to locate them. Participants will be trained on how to use each piece of the technology during their in-clinic sessions as well as during the equipment set up at their homes. They will also be provided with a take-home manual that clearly explains step-by-step guidelines for using the program. In case participants have difficulty connecting with the trainer during the Supervised Phase, the trainer will troubleshoot remotely by telephoning participants. 6) Our sample will represent older adult volunteers. The results will not be generalizable to younger amputees. We do not view this as a limitation given that > 80% of individuals with LLA in Western countries are older adults. The results will be limited to differences related to older volunteers. 7) Loss to follow up is a threat, particularly when participants are based remotely. To minimize loss to follow up, the site coordinators will maintain contact with participants once a month upon termination of the Unsupervised Phase under the premise of collecting information on falls.”

21) CONSORT: Generalisability (external validity, applicability) of the trial findings

21-i) Generalizability to other populations

21-ii) Discuss if there were elements in the RCT that would be different in a routine application setting

22) CONSORT: Interpretation consistent with results, balancing benefits and harms, and considering other relevant evidence

22-i) Restate study questions and summarize the answers suggested by the data, starting with primary outcomes and process outcomes (use)

This item is not relevant to the manuscript, because it is a protocol.

22-ii) Highlight unanswered new questions, suggest future research

Other information

23) CONSORT: Registration number and name of trial registry

“The study is registered with the Clinicaltrial.gov (NCT01942798).”

24) CONSORT: Where the full trial protocol can be accessed, if available

This item is not applicable because the manuscript is the protocol.

25) CONSORT: Sources of funding and other support (such as supply of drugs), role of funders

“This study is being funded by the Canadian Institutes of Health Research [MOP-130336], a grant from the Amputee Coalition of Canada and the University of Alberta-Franklin Fund. BI is supported by the Vanier Canada Graduate Scholarships.”

X26-i) Comment on ethics committee approval

x26-ii) Outline informed consent procedures

X26-iii) Safety and security procedures

X27-i) State the relation of the study team towards the system being evaluated
