# Supplementary material for: A Telehealth Intervention Using Nintendo Wii Fit Balance Boards and iPads to Improve Walking in Older Adults With Lower Limb Amputation (Wii.n.Walk): Study Protocol for a Randomized Controlled Trial
Source: JMIR Res Protoc. 2014 Dec 22;3(4):e80. doi: 10.2196/resprot.4031 (PMC4376145; doi:10.2196/resprot.4031)
Supplement: Supplementary file 2 [file resprot_v3i4e80_app2.pdf]

Canadian Institutes of Health Research/Instituts de recherche en santé du Canada

Notice of Recommendation/Avis de recommandation

Application Number/Numéro de la demande: 301139
Committee Code/Code du comité: SDA

Applicants/Candidats: Dr. William Cameron MILLER

With/Avec:

Mr. P. COX	Dr. A. DEATH	Dr. J. ENG
Dr. H. FINLAYSON	Dr. C. GOLDSMITH	Ms. B. IMAM
Prof. T. JARUS	Ms. L. MCLAREN	Mr. I. MITCHELL
Dr. M. PAYNE		

Institution paid/ Établissement payé: University of British Columbia

Title/Titre: Evaluation of the WiiFit to enhance walking in older adults with Lower Limb Amputation

Primary Inst./Inst. principal: Aging
Other Related Inst./ Autres inst. connexes: Musculoskeletal Health and Arthritis; Circulatory and Respiratory Health

Competition /Concours: Operating Grant
March/Mars 01, 2013

Number in competition/Nbre de demandes dans le concours: 2253

Peer Review Committee Recommendation, for your information and use/
Recommandation du comité d'examen par les pairs, pour fins d'information et d'utilisation:

**Committee/Comité:**	Social Dimensions in Aging
**Number reviewed/ Demandes examinées:**	33
**Application rank within the committee/ Rang de la demande dans le comité:**	4
**Percent Rank within the committee / Rang en pourcentage au sein du comité:**	12.12%
**Rated / Cote:**	4.21
**Recommended Term/ Durée recommandée:**	3 years/ans 0 months/mois
**Recommended average annual operating amount/ Montant annuel moyen recommandé pour le fonctionnement:**	$234,666
**Recommended equipment amount/ Montant recommandé pour les appareils:**	$0

This document is for information only.
An application rated below 3.50 is ineligible for CIHR funding. For applications rated 3.50 and above, please note that it is the application's rank within the peer review committee that determines whether it is funded, rather than its absolute rating. The final funding decision will be communicated in the Notice of Decision.

Document à titre d'information seulement.
Une demande cotée en dessous de 3,5 n'est pas admissible au financement des IRSC. En ce qui a trait aux demandes cotées 3,50 ou plus, veuillez noter que l'on détermine l'attribution des fonds en fonction du classement obtenu au sein du comité d'examen par les pairs plutôt qu'en fonction du classement absolu. La décision finale relative au financement sera communiquée dans l'Avis de décision.

Review Type/Type dévaluation: Committee Member 1/Membre de comité 1
Name of Applicant/Nom du chercheur: MILLER, William Cameron
Application No./Numéro de demande: 301139
Agency/Agence: CIHR/IRSC
Competition/Concours: 2013-03-01 Operating Grant/Subvention de fonctionnement
Committee/Comité: Social Dimensions in Aging/Dimensions sociales du vieillissement
Title/Titre: Evaluation of the WiiFit to enhance walking in older adults with Lower Limb Amputation

Assessment/Évaluation:

Canadian Institutes of Health Research

Operating Grant #301139

Miller: Evaluation of the WiiFit to enhance walking in older adults with Lower Limb Amputation

This application proposes to conduct a randomized clinical trial to evaluate the effectiveness of a Virtual Reality intervention for older adults with unilateral transtibial or transfemoral lower limb amputation. Participants will be randomized (blocked; stratified by site and level of amputation) to receive intervention using WiiNWALK, a home-based intervention targeting walking abilities, or the control intervention using Wii Big Brain, a cognitive intervention. Treatment will be provided in groups of 3 participants, 3 times per week for 4 weeks (supervised intervention) followed by a 4 week unsupervised period. Follow up outcome measurements will be conducted 1 year later. Outcomes include measures of walking capacity (2MWT), balance, gait speed and strength (Short Physical Performance Battery), physical activity (Physical Activity Scale for the Elderly), balance confidence (Activities-specific Balance Confidence Scale), life space mobility (Life Space Assessment) and health-related quality of life (Health Utility Index).

This well written proposal addresses an important question of how to provide cost-efficient rehabilitation intervention that is effective at maximizing mobility and is motivating to ensure adherence following LLA. The rationale is well stated and the literature review is comprehensive. Virtual Reality is a recent addition to rehabilitation interventions, and has been used with other populations (e.g. stroke, head injury, COPD) but is quite **novel** in this population. The use of a commercially available device means that this intervention, if found to be beneficial, may easily be integrated into the clinical services provided to clients with LLA. The authors made a strong case for the importance of addressing the problem of adherence related to the use of prescribed prosthetic devices, and therefore this study has **importance** in this clinical area.

The following suggestions relate to the **study methodology**:

1. The concepts related to the prevalence of abandonment (of the prosthesis) in this clinical population and the increased risk of falls is clearly stated. It would therefore be important to include measures of these constructs (i.e. abandonment and falls) to investigate whether the intervention has an impact on these outcomes. This would be appropriate to include due to the long follow up period of one year.

Review Type/Type dévaluation: Committee Member 1/Membre de comité 1
Name of Applicant/Nom du chercheur: MILLER, William Cameron
Application No./Numéro de demande: 301139
Agency/Agence: CIHR/IRSC
Competition/Concours: 2013-03-01 Operating Grant/Subvention de fonctionnement
Committee/Comité: Social Dimensions in Aging/Dimensions sociales du vieillissement
Title/Titre: Evaluation of the WiiFit to enhance walking in older adults with Lower Limb Amputation

Assessment/Évaluation:

2. It was stated in the proposal that the SAM (Orthocare Stepwatch Activity Monitor) will monitor the 'amount of physical activity performed in the community' (page 7). It is not clear how this device will differentiate between activity performed in the community and at home.

3. There is no information on the procedures that will be used to select and implement the outcome evaluators: who will the outcome evaluators be? how will they be trained? how will they be kept blind to the group assignment?

4. The intervention protocols are well described and well thought out. However, it is not clear how the groups of 3 participants will work together, specifically in relation to the component 'vicarious experience' (page 8). It is assumed that each of the participants will be functioning at different levels of mobility, will be engaging in different types of activities, and will be progressing at different rates. How will the sessions be conducted with these variations in clinical needs?

5. The progression of the intervention exercises during the unsupervised period is not described. While it would be most beneficial for the exercises to be modified according to each participant's progress, it is not clear if they will continue to perform the same exercises throughout this 4 week training period or whether there will be a mechanism to modify the intervention as the participant improves or regresses/has difficulties with the assigned exercises.

6. It is stated in the proposal that the Wii Big Brain group 'will be told to limit their non-scheduled use of the BigBrain at the end of each Supervised Phase session' (page 10). Will the participants in the WiiNWALK group also be asked to do the same? Also, it is not clear why they would be expected to not practice during the supervised period as one potential benefit of a home program is that clients can engage in the intervention multiple times. If this is included in the procedures, then the adherence to the intervention (total time and frequency of use) should be measured both during the unsupervised as well as the unsupervised period.

This is a strong **team of researchers** including experts in the field of rehabilitation, those with clinical expertise with clients with amputations, and members with the required methodological experience and expertise. There is support from the clinical milieus, making it very feasible that this project will be completed successfully.

Review Type/Type dévaluation: Committee Member 2/Membre de comité 2
Name of Applicant/Nom du chercheur: MILLER, William Cameron
Application No./Numéro de demande: 301139
Agency/Agence: CIHR/IRSC
Competition/Concours: 2013-03-01 Operating Grant/Subvention de fonctionnement
Committee/Comité: Social Dimensions in Aging/Dimensions sociales du vieillissement
Title/Titre: Evaluation of the WiiFit to enhance walking in older adults with Lower Limb Amputation

---

Assessment/Évaluation:

A. Candidats

The principal applicant, Dr Miller, has been a professor at British Colombia University, department of Occupational Therapy, since 2004. He has obtained several grants and distinctions considering his early career in research. He is a new investigator of the CIHR since 2007. Over the last 10 years, he supervised approximately 50 students, which most of them have published. He presently has to his credit a total of 100 articles, 7 book chapters and 188 abstracts. These articles are for the most part related to his field of research. This researcher is very active and his file is very solid and consistent with the research field proposed in this grant request.

Dr Ian Mitchell is an engineer as well as an associate professor at British Colombia University, department of occupational Science & Occupational Therapy since 2010. He has many grants as a principal investigator or co-investigator. He will be responsible for the technological and support aspects of this study.

Dr Tal Jarus, an occupational therapist, is a full professor at British Colombia University, department of Occupational Therapy. Essentially, she supervised non-thesis Master's students in occupational therapy. She will mainly contribute as a consultant on the issues related to virtual reality and gaming application.

Dr Janice Eng is a very productive researcher in rehabilitation. She has to her credit 160 articles, including over 12 rigorous randomised controlled trials in stroke rehabilitation. She received several prizes and contributed to enhancing the body of research in rehabilitation over the years. In this project she will contribute to the evaluation of homework-oriented rehabilitation.

Dr Charlie Goldsmith is a clinical epidemiologist and biostatistician at Simon Fraser University. He has an expertise in RCT design and will be responsible for the cost analysis.

Dr Heather Finlayson is a physician and clinical assistant professor at British Columbia University since 2010. She used to conduct randomised studies. She has experience in providing prosthetic rehabilitation.

Dr Payne is a physician and assistant professor at the University of Western Ontario. He also has extensive experience in providing prosthetic rehabilitation.

Two physiotherapists (L. MacLauren and P. Cox) complete the team. They are specialised in prosthetic rehabilitation and will be integrated for knowledge translation through special interest groups.

Review Type/Type dévaluation: Committee Member 2/Membre de comité 2
Name of Applicant/Nom du chercheur: MILLER, William Cameron
Application No./Numéro de demande: 301139
Agency/Agence: CIHR/IRSC
Competition/Concours: 2013-03-01 Operating Grant/Subvention de fonctionnement
Committee/Comité: Social Dimensions in Aging/Dimensions sociales du vieillissement
Title/Titre: Evaluation of the WiiFit to enhance walking in older adults with Lower Limb Amputation

---

Assessment/Évaluation:

There is no doubt that the principal researcher and the co-researchers have the required skills to accomplish the work they will undertake for the duration of this research grant.

B. Synopsis of the proposal

This team's grant proposal application aims to provide a rehabilitation program with commercially virtual reality gaming software, such as the WiiFitTM by Nitendo, to people with lower limb amputation (LLA). The hypothesis is that this approach is motivating and engaging as well as challenging and necessary because improvement requires highly repetitious task performance. The objective of the intervention is to optimize balance and lower limb strength, which in turn enhances functional mobility. The hypotheses are that compared to the control group the WiiNWALK group will: 1) experience an improvement in walking capacity, balance, gait speed, physical activity, balance confidence and life space mobility and quality of life; and 2) show no within or between group differences in total time and frequency of use between the supervised and unsupervised phases. Subjects in the control group will play cognitive oriented video games using WiiBig Brain Academy Degree Program (BigBrain^{tm}). To begin with, they developed software to enable WiiNWALK to operate the desired training program. A pilot study was done which pointed out issues relevant to the selection of meaningful control group activity in order to minimize lost to follow-up. Then, a feasibility pilot study was conducted with six participants and showed statistical improvement in walking capacity, balance and leg strength. To achieve their research objective, the authors will use a randomised controlled trial to evaluate the effect of the WiiNwalk program in 72 older (50+ years) community living adults who have a unilateral above or below knee amputation. Randomisation will occur after the participant's screening and the completion of the baseline measurements. All clinic and home sessions will be supervised by an expert and conducted in groups of 3. Visual contact between the trainer and the subjects will be maintained using tablet technology. The subjects will be evaluated: 1) before the intervention, 2) 6 weeks and 11 weeks later (after an unsupervised phase (weeks 7 -10)), 4) and 63 weeks later to evaluate the effect of retention. Both groups will receive 40 minutes of supervised group exercises, 3 times per week for a period of 4 weeks. Intervention conducted over the first week will be held at the clinic, while the remaining three weeks will be conducted at the subject's home with an electronic tablet. At the end of the supervised interventions (4 weeks), subjects will retain the Wii units and will be encouraged to use the program on their own for an additional 4-week period (without supervision).

C. Assessment of the proposal

Significance:

Evidence suggested that those who are most likely to reject the use of their prosthesis are older adults and

Review Type/Type dévaluation: Committee Member 2/Membre de comité 2
Name of Applicant/Nom du chercheur: MILLER, William Cameron
Application No./Numéro de demande: 301139
Agency/Agence: CIHR/IRSC
Competition/Concours: 2013-03-01 Operating Grant/Subvention de fonctionnement
Committee/Comité: Social Dimensions in Aging/Dimensions sociales du vieillissement
Title/Titre: Evaluation of the WiiFit to enhance walking in older adults with Lower Limb Amputation

---

Assessment/Évaluation:

those with TF amputation. Abandonment can lead to decreased functional mobility and increased dependence. Thereby, the proposed population in this request is pertinent as well as being a clinical reality.
Research Plan
This project was built from previous experience and results obtained from previous works in addition to a pilot study conducted by the team. The different study propositions for each objective are easy to understand. The statistical analyses of each theme explored are well presented. By reading this section, I am convinced that the researchers, based on earlier results, will be able to conduct the necessary analyses to attain the proposed objectives. Clinical measures are pertinent and demonstrated good metric quality, except for the PASE, which would probably be modified or used partially (I am not sure that it can be used with the targeted population). Because there are two recruitment sites, the recruitment seems possible in the timeline.

However, I do need further explanation on some aspects of the study. For the safety, another person will be at home during the program exercises? Furthermore, concerning the motivation, how can the researchers be sure that collecting information on falls will not influence the motivation to do exercises during the unsupervised phase?
Originality:

The rational of the proposal is based on the observation that older individuals with LLA are often discharged prior to reaching sufficient levels of prosthetic function. This early discharge problem is one of the principal reasons for high rates of prosthesis abandonment in this age group. The ultimate objective of this innovative approach is to improve outcomes post discharge. It is true that Virtual reality (VR) gaming technologies is presented as an excellent tool that may translate to self-managed or monitored home therapies in older adults. However, these results are not demonstrated with this particular, challenging population (and are not easy to demonstrate because of the presence of multiple co-morbidity). Also, it is still important to document home treatments in this population because they have limited access to facilities and transportation. Furthermore, in virtual rehabilitation, they used a home-rehabilitation group which is particularly new and useful.

Research Environment:

The principal researchers of this grant proposal showed that the team can conduct the proposed study. Their collaboration seems well established. Clinicians have sustained the project since the beginning. Furthermore, two sites will facilitate the recruitment particularly for this population.
In summary, the procedure is well written and well described, part of the methodology has already been verified and means have been taken to ensure that the results are pertinent. The population seems accessible but the author must specify how they will be sure to get the number in the timeline (they have to be careful

Review Type/Type dévaluation: Committee Member 2/Membre de comité 2
Name of Applicant/Nom du chercheur: MILLER, William Cameron
Application No./Numéro de demande: 301139
Agency/Agence: CIHR/IRSC
Competition/Concours: 2013-03-01 Operating Grant/Subvention de fonctionnement
Committee/Comité: Social Dimensions in Aging/Dimensions sociales du vieillissement
Title/Titre: Evaluation of the WiiFit to enhance walking in older adults with Lower Limb Amputation

Assessment/Évaluation:

with the exclusion criteria).
Based on the fact that this is a pertinent study of a major research problem, that the methodology is well presented, and, lastly that the authors have published some results of the work related to this grant, I find this proposal excellent.

Budget

This budget is fair and reasonable. I do not see any superfluous expense.

Review Type/Type dévaluation: SO Notes /Notes de l'agent scientifique
Name of Applicant/Nom du chercheur: MILLER, William Cameron
Application No./Numéro de demande: 301139
Agency/Agence: CIHR/IRSC
Competition/Concours: 2013-03-01 Operating Grant/Subvention de fonctionnement
Committee/Comité: Social Dimensions in Aging/Dimensions sociales du vieillissement
Title/Titre: Evaluation of the WiiFit to enhance walking in older adults with Lower Limb Amputation

---

Assessment/Évaluation:

Miller

The research environment is very good and the team has all the necessary expertise to conduct the research project.

This is a very well written proposal in an important area, and it concerns a novel application. The intervention can be self motivating, can provide practice opportunities, and is cost effective. The selection of the control group was appropriate. The intervention also follows a logical progression, and the key components of the methodology have been verified

The applicant should discuss the outcome evaluators in more detail, including selection, training and how blind will be maintained. Other participation obstacles such as falls could have been included as outcome measures. The application also could have had more detail on the implementation of the group intervention since the pilot did not encompass a group aspect. Pace may not be able to be fully gauged with the intervention. In the analysis, ANCOVA discussion should have included a list of controls.

No budget concerns raised.

No ethical issues raised.
